# Evolution and developmental functions of the dystrophin-associated protein complex: beyond the idea of a muscle-specific cell adhesion complex

**DOI:** 10.3389/fcell.2023.1182524

**Published:** 2023-06-13

**Authors:** Vincent Mirouse

**Affiliations:** Institute of Genetics, Reproduction and Development (iGReD), Université Clermont Auvergne-UMR CNRS 6293-INSERM U1103, Faculté de Médecine, Clermont-Ferrand, France

**Keywords:** basment membrane, f-actin, evolution, morphogenesis, muscle

## Abstract

The Dystrophin-Associated Protein Complex (DAPC) is a well-defined and evolutionarily conserved complex in animals. DAPC interacts with the F-actin cytoskeleton via dystrophin, and with the extracellular matrix via the membrane protein dystroglycan. Probably for historical reasons that have linked its discovery to muscular dystrophies, DAPC function is often described as limited to muscle integrity maintenance by providing mechanical robustness, which implies strong cell-extracellular matrix adhesion properties. In this review, phylogenetic and functional data from different vertebrate and invertebrate models will be analyzed and compared to explore the molecular and cellular functions of DAPC, with a specific focus on dystrophin. These data reveals that the evolution paths of DAPC and muscle cells are not intrinsically linked and that many features of dystrophin protein domains have not been identified yet. DAPC adhesive properties also are discussed by reviewing the available evidence of common key features of adhesion complexes, such as complex clustering, force transmission, mechanosensitivity and mechanotransduction. Finally, the review highlights DAPC developmental roles in tissue morphogenesis and basement membrane (BM) assembly that may indicate adhesion-independent functions.

## Introduction: dystrophin and dystrophin-associated protein complex, a story linked to human genetic diseases

**FIGURE 1:**
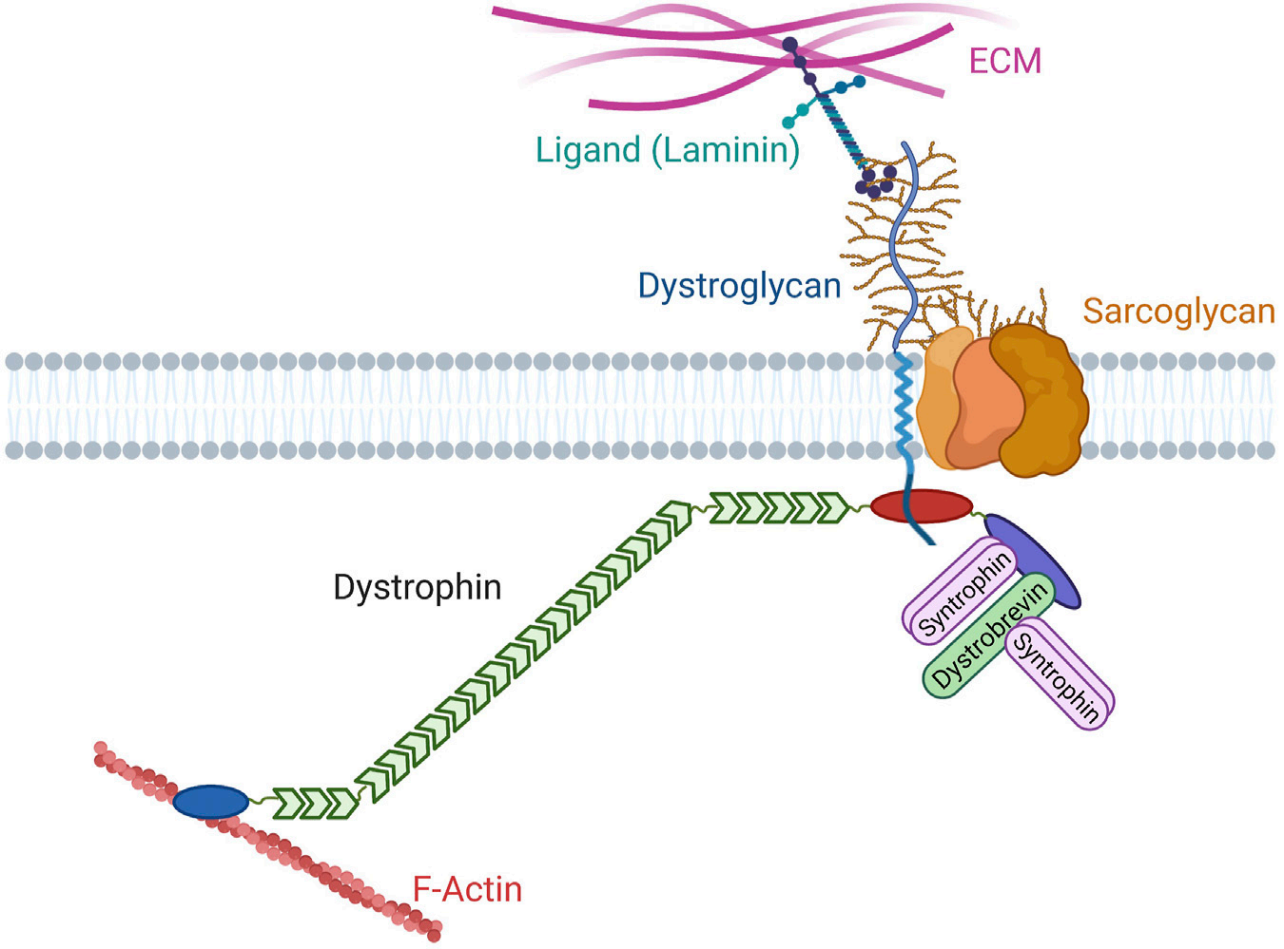
Canonical organization of the Dystrophin Associated Protein Complex. Dystroglycan (blue) is a glycosylated (brown) protein that interacts with extracellular matrix (ECM) proteins containing laminin globular domains, such as laminin. Its intracellular extremity interacts with dystrophin. Dystrophin contains an actinin-like actin biding domain (blue), a rod domain with 24 spectrin repeats (green), several motifs for the interaction with dystroglycan (red), and a C-terminal domain (purple) that harbors two motifs for binding to syntrophins (pink) and a motif for binding to dystrobrevin (green). Dystrobrevin also contains two syntrophin binding motifs. Different subunits of the proteoglycan sarcoglycan are also found in the complex.

Dystrophin was initially identified through the discovery of the gene involved in Duchenne Muscular Dystrophy (DMD), a severe muscle disease (Hoffman et al., 1987). Since then, dystrophin has been extensively studied to understand DMD etiology and to develop therapeutic approaches. For instance, the identification of dystrophin point mutations or internal deletions in patients with DMD or Becker Muscular Dystrophy (BMD), a milder form of the disease, has provided insights into the relevance of the different dystrophin protein domains. Then, additional proteins were identified as part of the same protein complex, using biochemical approaches on muscle extracts (e.g., dystroglycan, dystrobrevin, syntrophins, and sarcoglycans) (Ervasti and Campbell, 1991) (Figure 1). Dystroglycan and sarcoglycans are transmembrane glycoproteins while dystrobrevin and syntrophins are cytosolic proteins. Dystrophin directly interacts with dystroglycan, syntrophins and dystrobrevin, placing it at the heart of this complex (Suzuki et al., 1992; Ahn and Kunkel, 1995; Suzuki et al., 1995; Sadoulet-Puccio et al., 1997). Some of these proteins are also implicated in other genetic disorders associated with muscle defects (Kanagawa, 2021; Vainzof et al., 2021). Together, these proteins form the Dystrophin-Associated Protein Complex (DAPC) or dystrophin glycoprotein complex. It was quickly established that dystrophin binds to the F-actin cytoskeleton and that dystroglycan is a receptor for laminin, an extracellular matrix (ECM) protein (Levine et al., 1990; Ibraghimov-Beskrovnaya et al., 1992) (Figure 1). DMD-associated muscle fragility and the general similarity between DAPC and the well-characterized integrin complex have led to the prevalent view that DAPC provides a permanent mechanical link between cytoskeleton and ECM by allowing a strong adhesion between these molecular structures. However, despite some macroscopic observations in favor of adhesive properties, molecular evidence to validate/invalidate this view is lacking. Indeed, classical genetic models in mammals and studies in patients have been valuable to understand the general pathophysiology, but they have limitations for state-of-the-art cell biology approaches. For comparison purposes, the adhesive properties of integrins have been thoroughly characterized, mainly using different cell types in cell culture systems and invertebrate models. Then, this knowledge was transferred to the muscle context. For instance, the integrin complex mechanosensitive properties were demonstrated a long time ago, but only recently they have been confirmed *in vivo* in *Drosophila* muscles, and not in vertebrates yet (Lemke et al., 2019). Likewise, despite decades of studies, DAPC key functions and properties and consequently DMD molecular etiology remain unclear. Nonetheless, as detailed below, DAPC is not restricted to muscle cells. Thus, data coming from other tissues may be valuable to understand the molecular functions of this conserved complex.

Complementary to experimental data, studying DAPC evolution can be very precious to obtain insights into the relevant aspects of its function. In this review, we will first discuss DAPC origin and diversification and focus on dystrophin evolution and compare it with the current knowledge on its functional domains. Then, we will systematically compare DAPC with well-defined adhesion complexes to determine whether it shares their common features. Lastly, we will illustrate DAPC developmental functions that may help to understand its roles in muscle cells.

## Evolution of the dystrophin-associated protein complex

### Appearance and diversification of DAPC components

**FIGURE 2:**
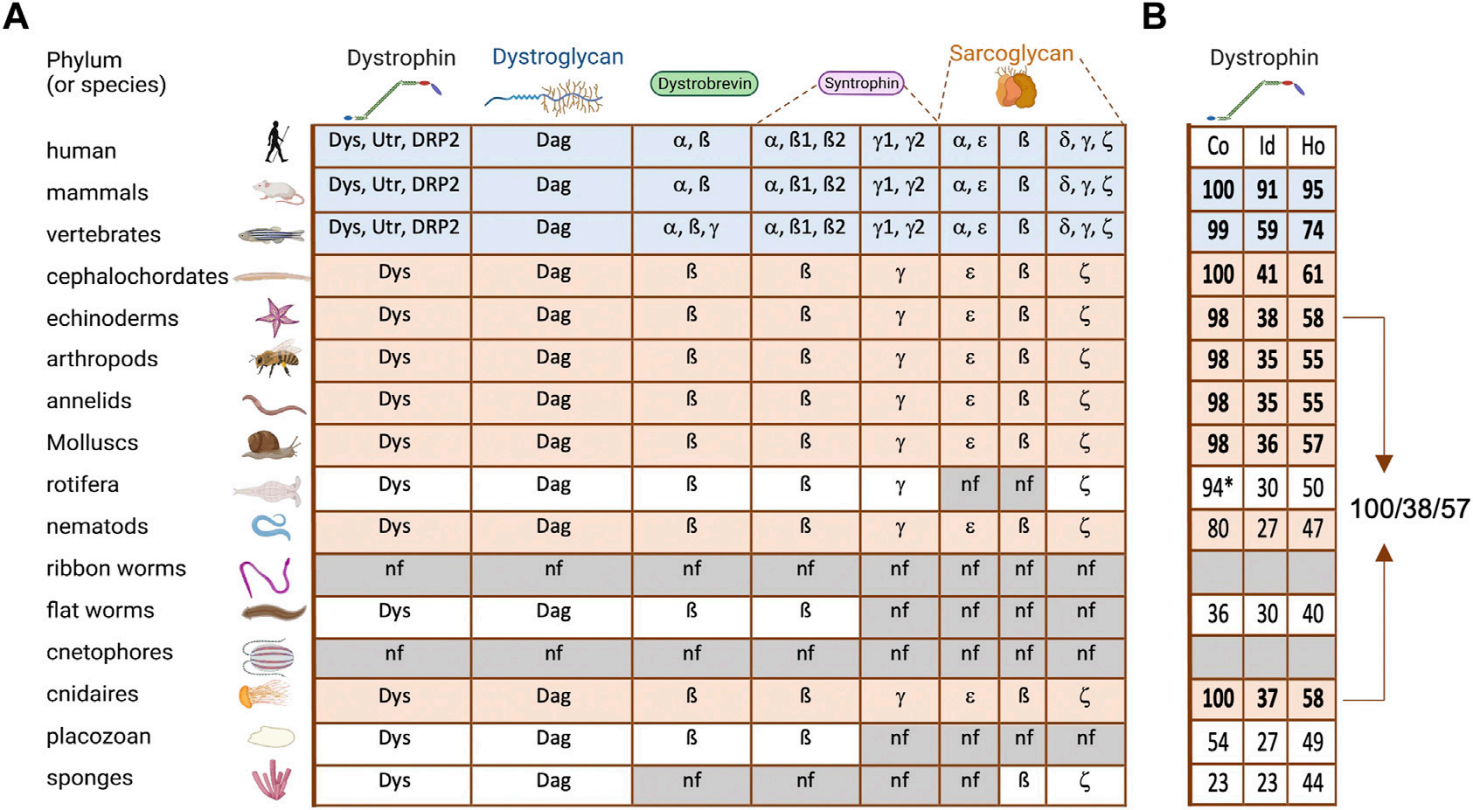
DAPC evolution in metazoans **(A)** On the left are listed and illustrated the main phyla of metazoan evolution. The table contains all the DAPC component paralogs (Dys, dystrophin; Utr, utrophin; DRP2, dystrophin related protein 2; Dag, dystroglycan). Blue lines: all vertebrates analyzed, revealing an important diversification. Orange lines: invertebrates with a complete DAPC. **(B)** Percentage of alignment coverage (Co), amino acid identity (Id) and homology (Ho) between the human dystrophin sequence (Dp427m isoform) and its orthologs with the best score in each phylum. In bold are the ones kept for Figure 3. *this coverage was obtained with two distinct proteins.

DAPC is absent in the genomes of bacteria and plants, but present in the genomes of most species of the animal kingdom. Indeed, dystroglycan and dystrophin are found in all metazoans, except for ctenophores, but not in unicellular organisms (Adams and Brancaccio, 2015). Reinvestigation of the currently available genomic data globally confirmed these conclusions, but also suggests that DAPC is absent in *Nemertea* (ribbon worms) (Figure 2A). This observation is striking because it means that dystrophin and dystroglycan are already present in sponges, thus before the appearance of muscle cells (Steinmetz et al., 2012). Conversely, ctenophores and *Nemertea* have muscle cells, but no DAPC. Hence, DAPC appearance and maintenance are not intrinsically linked to muscle. It also indicates that these two DAPC core components appeared “simultaneously” and have always coevolved.

Analysis of the presence of other DAPC components in the same genomes revealed that some sarcoglycan classes are present in sponges, while syntrophin and dystrobrevin only appeared in our common ancestor with cnidarians (Figure 2A). Interestingly, syntrophins are already diversified in two classes in this phylum: beta-syntrophin and gamma-syntrophin. As described previously, dystrobrevin is probably a very old paralog of dystrophin (Jin et al., 2007). Notably, dystrobrevin is known to have, as dystrophin, two binding sites for syntrophins [(Newey et al., 2000) and Figure 1]. However, a study revealed a complex structure of dystrobrevin genes in most tetrapods excepted murids, with alternative splicing events leading to an absence or a swap of the first of these binding sites (Böhm et al., 2009).

This analysis also indicates that the important diversification observed in DAPC components, except for dystroglycan, only occurred in vertebrates. Such diversification can be a source of redundancy but may also explain the separation of exiting functions and the appearance of new ones. For instance, alpha-syntrophin and beta-syntrophin have the same origin, but they have evolved asymmetrically, and currently alpha-syntrophin is much more different from their common ancestor. This suggests the acquisition of specific new functions. Similarly, all invertebrate dystrophins are closer to human dystrophin than to its paralog utrophin. In vertebrates, dystrophin expression is found in the nervous system and in the muscle cell lineage from their stem cells (satellite cells) to differentiated myofibers while epithelial tissues expressed utrophin (Hoffman et al., 1988; Durbeej and Campbell, 1999; Dumont et al., 2015). Accordingly, dystrophin is also found in epithelia in invertebrates, which does not contain dystrophin paralog in their genome (Cerqueira Campos et al., 2020). Furthermore, it is important to keep in mind that alternative promoters and splicing represent another important level of diversification, leading to multiple isoforms for a single gene. This is especially true for dystrophin, for which many isoforms with different expression patterns have been identified, although this point will not be discussed in this review.

### Dystrophin evolution

#### Human dystrophin secondary structure is already found in cnidarians

**FIGURE 3:**
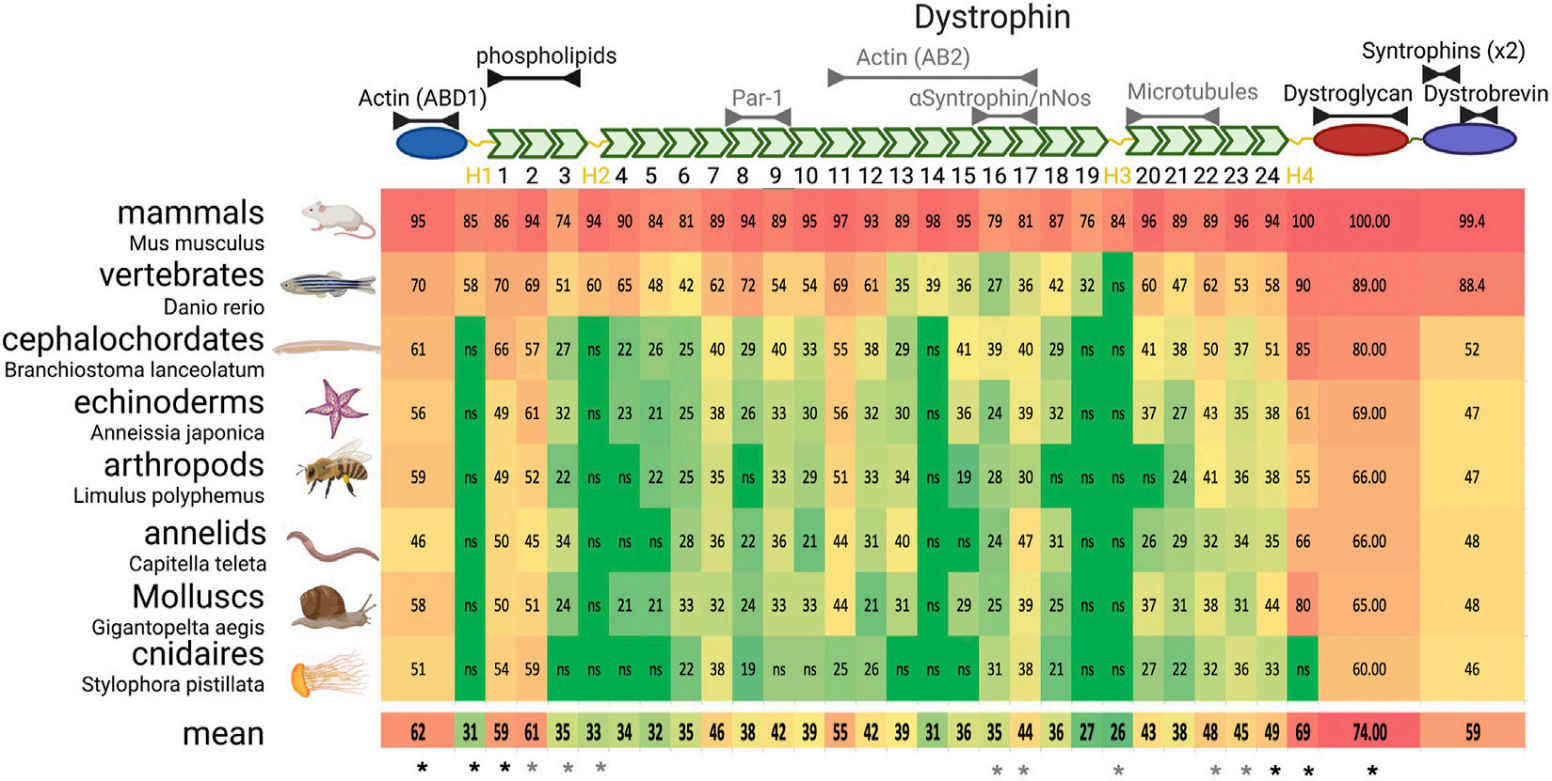
Dystrophin domain evolution in metazoans. Upper panel) Schematic representation of dystrophin structure. From the left (N-terminal) to the right are found the actinin-like domain, the rod domain with its spectrin repeats (green) and hinges (yellow), the dystroglycan interacting domain and the C-terminal domain. Interactions likely (black) or unlikely conserved (grey) in invertebrate dystrophin are indicated above the domains. (Lower panels) On the left are listed and illustrated the phyla with the name of the exact species analyzed. Of notice, the drawn animal is representative of the phylum and not necessarily of the analyzed species. The table indicates the percentage of identity with each domain of human dystrophin, color-coded in function of the identity percentage (from green to red). As the limit of significant identity detection was 19%, 18% was retained to determine the mean conservation if the alignment was not significant (ns). * indicate the domains that are systematically (black) or sometimes (grey) used in therapeutic microdystrophins.

Some studies have precisely analyzed dystroglycan evolution and suggest that its binding to the ECM through its extracellular domain and to dystrophin through its intracellular domain are constant properties (Adams and Brancaccio, 2015; Brancaccio and Adams, 2017). Dystrophin evolution appears more complex. Before discussing its evolution, it is important to describe the secondary structure of the canonical muscle long isoform of human dystrophin (encoded by the Dp427m transcript variant) (Figure 3). The N-terminal part includes an actin binding domain (ABD) similar to the one of actinin and composed of two calponin homology domains. This is followed by a very long rod domain composed of 24 spectrin repeats (SRs). Each SR is made of ∼100 amino acids forming three alpha helixes that potentially fold on each other. Then, there is a WW domain and a cysteine-rich domain containing two EF-Hand motifs and a zing-finger-like ZZ domain. The region from the WW domain to the ZZ domain is necessary and sufficient for efficient binding to dystroglycan. Finally, the C-terminal domain is involved in interactions with dystrobrevin and syntrophins via distinct motifs.

In sponges, the longest dystrophin (found in *Amphimedon queenslandica*) includes only six SRs, with a clear homology with human dystrophin starting at SR20, and the dystroglycan interacting domain. Then, probably through fusion with a spectrin gene, dystrophin acquired its complete secondary structure in the common ancestor of cnidarians and other metazoans [Figure 2 and (Jin et al., 2007)]. This structure is found in almost all subsequent phyla, although it diverged more in some phyla, or even seems to have been split in two proteins, such as in *Rotifera*.

Importantly, in all phyla harboring a complete dystrophin, the identity and homology percentages tend to be similar (Figure 2B). For instance, human dystrophin displays 58% and 55% of homology with its echinoderm and cnidarian orthologs, respectively, which are two very distant phyla. These scores might be explained by a fast and recent evolution in chordates. However, echinoderm and cnidarian dystrophin share 57% of homology (Figure 2B). This suggests a conserved minimal sequence for the whole protein with ∼35% of identity and 55% of homology through at least 600 million years of evolution.

#### Dystrophin domains evolve at different rates

Although dystrophin roles in humans might rely on functions newly appeared during evolution, it is reasonable to think that conserved domains may be the most important. Arguing for such a logic, dystrophin is required for muscle maintenance during adult life also in invertebrate models (e.g., *Drosophila*) (Shcherbata et al., 2007; van der Plas et al., 2007). Importantly, such functional conservation at the tissue scale in evolutionarily divergent species authorizes to focus on what is conserved at the molecular scale, but also to eliminate none conserved domains as main contributors of DMD etiology. The conserved domains might also be informative about relevant partners.

Thus, a systematic alignment was performed, domain by domain, between human dystrophin and the best match in each phylum with a full-length coverage of the alignment to facilitate the analysis (Figure 3). Although some phyla were excluded from this approach, it covered a very large proportion of animals, from mammals to cnidarians. This alignment showed that the actinin-like domain (ABD1) and dystroglycan-interacting domain are very conserved, in agreement with their critical role in dystrophin, and will not be discussed here.

Some parts of the C-terminal domain are very well conserved, but not all. For instance, several studies identified two motifs (positions 3,432–3,445 and 3,467–3,479) that interact with syntrophins and found the same motif also in dystrobrevin and utrophin (Suzuki et al., 1995; Yang et al., 1995; Newey et al., 2000). Another study mapped the interaction with dystrobrevin in the adjacent short coiled-coil domains (3,496–3,530 and 3,555–3,600) (Sadoulet-Puccio et al., 1997). Both syntrophin-interacting motifs and dystrobrevin-binding coiled-coil domains are extremely well conserved in all species where these proteins are found. Conversely, the other parts of the C-terminal domain are very poorly conserved. This strongly suggests that dystrophin C-terminus has no other conserved function than the recruitment of these two protein classes.

Lastly, the rod domain is probably the one for which adopting an evolutionary perspective is most relevant. The first obvious finding from its phylogenetic analysis is that the selective pressure is highly variable from one SR to another, suggesting that the SRs are not equivalent. This also confirms a general conclusion from mutation analyses in patients with BMD and structure-function analyses in the mouse, as discussed in the next paragraphs on SR function and conservation.

#### SR1-3: interaction with phospholipids

SR1 and SR2 are the two most evolutionarily conserved SRs (Figure 3). Structure-function analyses confirmed their relevance in muscle, explaining why at least one of these SRs is systematically included in all current therapeutic versions of microdystrophin (see below). For instance, SR2 and 3 are important to maintain muscle force after repeated eccentric contractions (Nelson et al., 2018). The SR1-3 region interacts with phospholipids and their expression is sufficient for recruitment to the sarcolemma (Legardinier et al., 2008; Zhao et al., 2016). It is generally proposed that dystrophin lies on the cell membrane, with the N-terminus part of the rod domain associated with cell membrane and the dystroglycan interacting domain close to the C-terminus. However, it is not known yet why the interaction with phospholipids is critical for dystrophin function. Moreover, it is unclear whether this interaction explains the strong SR1-2 conservation or whether it reflects additional interactions and functions that remain to be identified.

#### SR8-9: interaction with MARK2/Par-1b and satellite cell asymmetric division

Both dystrophin and utrophin interact with the cell polarity kinase MARK2/Par-1b (Yamashita et al., 2010). This interaction is thought to be crucial for Par-1 recruitment to one side of the asymmetrically dividing satellite cells (muscle stem cells) (Dumont et al., 2015). Mirroring its function in invertebrates, Par-1 then excludes Par-3 to the other cell side, resulting in asymmetric division (St Johnston and Ahringer, 2010). *In vitro* data indicate that this interaction requires both SR8 and SR9, although only SR9 is conserved in evolutionarily distant species (Figure 3). Thus, this interaction relies on poorly conserved motifs, or the interaction is not conserved. In the second hypothesis, a functional explanation for the strong conservation of SR9 is needed. Notably, these SRs have never been specifically included in microdystrophins. Therefore, it is unclear whether this interaction is important to explain DMD etiology, particularly for muscle regeneration, and whether they present a therapeutic interest. Similarly, the muscle regenerative capacities in patients with BMD with or without SR8-9 have not been compared yet.

#### SR11-14 or SR11-17: the second actin binding domain (ABD2)

*In vitro* biochemical studies have identified a second ABD within the rod domain (ABD2) (Rybakova et al., 1996). More investigations defined two fragments starting at SR11 and finishing at SR14 or SR17 with comparable affinity for actin (Kd = 14 and 7 μM, respectively) (Amann et al., 1998; Amann et al., 1999). This whole region is basic, a characteristic feature of ABDs. It has been proposed that the association of the two distant ABDs lies dystrophin along F-actin and that a single dystrophin molecule is associated with an actin filament portion of more than 20 monomers (Rybakova and Ervasti, 1997). Interestingly, in utrophin, this region is not basic, except for SR11, and does not bind to actin, despite the strong homology with dystrophin. Strikingly, SR11 is the most conserved SR in evolution after SR1-2, indicating a strong selective pressure (Figure 3). Conversely, the downstream sequences (SR12-17) are poorly conserved, and their isoelectric point is acidic (∼5) in all non-vertebrate dystrophins. Thus, although the significance of SR11 conservation is unclear, it is likely that ABD2 is absent in invertebrates. Interestingly, expression of a construct that includes the region from SR10 to SR12 is partially targeted to the skeletal but not the cardiac sarcolemma, suggesting that it may interacts with a cortical protein that is differentially expressed in these two tissues (Zhao et al., 2016).

#### SR16-17: interaction with alpha syntrophin and recruitment of neuronal nitric oxide synthase

Dystrophin is necessary for the sarcolemmal localization of neuronal nitric oxide synthase (nNOS) (Brenman et al., 1995). The mechanism of recruitment has been debated. Indeed, nNOS binds directly to the PDZ domain of alpha-syntrophin and its localization is disrupted in alpha-syntrophin mutants (Hillier et al., 1999). Syntrophins interact with dystrophin C-terminus directly or through dystrobrevin (Figure 1). However, dystrophin C-terminus is not sufficient to recruit nNOS (Lai et al., 2009). Moreover, nNOS localization at the sarcolemma requires part of the rod domain, specifically SR16-17 (Lai et al., 2009; Lai et al., 2013). It was initially proposed that SR16-17 and nNOS directly interact, based on yeast two-hybrid assay results (Lai et al., 2009). However, another study proposed that dystrophin binds first to alpha-syntrophin via a motif like the ones present in the C-terminus and then indirectly recruits nNOS (Adams et al., 2018). These data reconcile the dual requirement of alpha-syntrophin and SR17 in nNOS recruitment. Moreover, a microdystrophin containing SR16-17 cannot rescue nNOS sarcolemmal localization in a double dystrophin and alpha-syntrophin mutant mouse (Adams et al., 2018).

The importance of nNOS recruitment by dystrophin also can be discussed. It has been proposed that nNOS recruitment promotes vasodilation in muscle tissue during exercise and prevents ischemia (Sander et al., 2000; Thomas et al., 2003; Kobayashi et al., 2008). However, alpha-syntrophin knock-out in the mouse blocks nNOS recruitment, but does not lead to muscle defects (Kameya et al., 1999; Thomas et al., 2003). Hence, the absence of nNOS recruitment is not the main cause of muscle fragility, but may exacerbate muscle degeneration in a context where other dystrophin functions are affected. Accordingly, adding SR16-17 to microdystrophin restores nNOS recruitment and improves the efficiency of such constructs (Duan, 2018; Boehler et al., 2023; Chamberlain et al., 2023). Additionally, a study in patients with BMD showed a correlation between the disease severity and nNOS absence at the sarcolemma (Gentil et al., 2012).

From an evolutionary perspective, SR17 is well conserved, but not SR16 (Figure 3). The precise mapping of the motif required for nNOS recruitment identified the very beginning of SR17 first helix. This motif is supposed to bind directly to alpha-syntrophin based on similarities with dystrophin C-terminus (Lai et al., 2013; Adams et al., 2018). However, despite SR17 conservation, this motif is poorly conserved, even in the mouse. Moreover, some data indicate that the two last helices of SR16 also are involved, although their conservation is very low. This suggests that the consensus motif for syntrophin binding is very loose, or that this interaction appeared recently during evolution. As mentioned before, alpha-syntrophin exists only in vertebrates. Moreover, invertebrate genomes do not have the nNOS isoform encoding the motif involved in the interaction of its mammal homolog with syntrophin PDZ domain (Hillier et al., 1999). Therefore, it seems very likely that the functional link between nNOS and the DAPC is limited to vertebrates or even to some mammals. Consequently, the reason for the good conservation of SR17 is not explained by the available functional data.

#### SR 20-22: interaction with microtubules

Microtubules are disorganized in the myofibers of *mdx* mice (dystrophin mutant). This observation led to the demonstration that dystrophin interacts directly with microtubules (Percival et al., 2007; Prins et al., 2009). It was initially proposed that SR24 was involved in this interaction. However, subsequent work indicated that only SR20-22 are required but not sufficient for the interaction with microtubules, suggesting a complex link (Prins et al., 2009; Belanto et al., 2014; Belanto et al., 2016). However, dystrophin capacity to rescue the microtubule defect in myofibers is independent of this domain (Belanto et al., 2016). In fact, the microtubule network disorganization in *mdx* myofibers might reflect a secondary consequence of the high regenerative activity in this context (Randazzo et al., 2019). Accordingly, the rescue of the degenerative process in *mdx* mice by pharmacological treatment or by dystrophin transgenes correlates with restoration of the normal microtubule organization (Percival et al., 2007; Belanto et al., 2014; Belanto et al., 2016; Nelson et al., 2018; Osseni et al., 2022). In terms of evolution, SR20-24 form a block of well-conserved SRs with a mean identity of 44% between human and all the considered species (Figure 3) and of 34% between humans and invertebrates only. However, these SRs are also well conserved between utrophin and invertebrate dystrophin (32%), although utrophin cannot bind to microtubules (Belanto et al., 2014). In conclusion, the potential role of a direct interaction between mammal dystrophin and microtubules remains to be identified, and the available functional data cannot explain SR20-24 conservation throughout evolution.

#### Hinges

Four hinge regions, one at each extremity and two at different internal positions of the rod domain, have been identified. These regions do not display a clear secondary structure or known motifs, and are enriched in proline, suggesting that they correspond to unfolded regions. Structure-function analyses performed in the context of microdystrophin optimization indicated that they are important, although to a different extent, and that their relationship with SRs can be complex ((Banks et al., 2010) and below). Their function could be to isolate distinct subdomains for promoting their correct conformation and/or for giving enough flexibility at specific places. The sequences of these regions are very poorly conserved, except for H4, possibly due to its proximity to the dystroglycan-binding domain, further supporting the idea that they do not contain a specific motif for a particular function.

#### Highly conserved spectrin repeats without a clear function or interactor

The previous paragraphs indicate that the selective pressure on some SRs rather than on others can hardly be explained by our current understanding of their function in most of the cases. Some well conserved SRs, such as SR7, have never been involved in any specific function or interaction. For others, such as SR11, SR17, and SR20-24, the available functional and biochemical data do not bring information on their conservation. For SR9, it would be interesting to determine whether the interaction with Par-1 is a conserved feature in invertebrates, to explain its conservation. Similarly, SR1-2 conservation may be explained by their interaction with phospholipids, but it is still unknown why such interaction is required for dystrophin function. Altogether, molecular mechanisms still need to be explored to explain the conservation of some SRs from corals to humans.

#### Comparison of conserved domains and therapeutic microdystrophin proteins

Gene therapy approaches using adeno-associated virus (AAV) vectors are limited by the DNA size that can be packed (5 Kb at most). As dystrophin cDNA is > 11 kb in size, much effort has been focused on testing shorter versions of dystrophin cDNA (i.e., microdystrophins) to rescue dystrophin activity in skeletal and cardiac muscles. Various microdystrophin versions currently coexist and have reached the critical step of clinical trials. Several reviews explain the rationale of their design (Duan, 2018; Boehler et al., 2023; Chamberlain et al., 2023). This is mainly based on the in-frame deletions observed in patients with BMD and removing parts of the rod domain, and on subsequently testing various constructs in murine (or canine) DMD models. The main conclusions are that the C-terminal domain is dispensable and that large parts of the rod domain can be deleted. Then, the main challenge is to find the best combination of 4–5 SRs. However, this approach presents at least three limitations. First, possible internal in-frame deletions due to dystrophin gene structure do not allow obtaining insights into the requirement of some SRs. Second, for most SRs, the deletions found in patients with BMD do not provide specific data on single SR because usually more than one SR is deleted. Third, deletions, depending on their breakpoints, can affect the protein conformation and stability, thus complicating the result interpretation. Ideally, a precise understanding of the function of the different SRs is required to really improve this rationale. So far, the only clear contribution coming from functional data is the incorporation of SR16-17 to recruit nNOS.

In the absence of enough functional data, conservation during evolution could be a good indicator to test new constructs and/or to orient functional studies towards the most conserved regions. Also, an interesting perspective could be to design chimeric SRs that associate relevant features. However, such approach might be delicate because of conformational issues. The descending order based on homology conservation during evolution, limited to the top ten, is SR2 > SR1 > SR11 > SR24 > SR22 > SR7 > SR23 > SR17> SR20 > SR9. For comparison, the microdystrophins currently tested in clinical trials systematically contain SR1 and SR24 and different combination of SR2, SR3, SR16, SR17, SR22, and SR23 (Figure 3). To our knowledge, the therapeutic interest of SR11 and SR7 has never been assessed.

A slightly different approach could be used for the hinges. H1 and H4 are always included, whereas the necessity of H2 and H3 is less obvious. Interestingly, some species have a significantly shorter H1 (e.g., 33 amino acids in echinoderms *versus* 95 in humans). Their use instead of the human sequence may allow gaining space to include other more relevant sequences.

Although several microdystrophins are currently tested in patients, their comparison with dystrophin evolution suggests that there is still room for optimization. Moreover, better understanding the functional role of each dystrophin conserved domain might be important to predict the efficiency of other therapeutic strategies, such as exon-skipping.

## Is DAPC a cell adhesion complex?

### Macroscopic and indirect evidence for a mechanical role

Various definitions have been proposed to explain what is considered the “mechanical function” of dystrophin and the DAPC, such as “shock absorber,” “to provide mechanical resistance to the sarcolemma,” or “to link the intracellular cytoskeleton to the ECM and transmitting forces” (Le et al., 2018; Dowling et al., 2021; Chamberlain et al., 2023). All these definitions implicitly assume that the DAPC can mediate a strong adhesion. The hypothesis of DAPC mechanical role is based on two main observations. The first is the sarcolemma fragility observed in patients with DMD and animal models of the disease. This might be the main reason of muscle degeneration. Importantly, sarcolemma rupture is caused by the mechanical stress associated with muscle contraction (Petrof et al., 1993). The second one is the discovery that dystroglycan binds to the ECM, while dystrophin binds to F-actin (Levine et al., 1990; Ibraghimov-Beskrovnaya et al., 1992). This led to the tempting hypothesis that the cytoskeleton-ECM connection created by the DAPC directly protects muscle cells from mechanical damage. Moreover, dystroglycan interaction with its ECM ligands and dystrophin interaction with F-actin are critical for DAPC function in muscle cells. This hypothesis is further supported by the findings that dystroglycanopathies are associated with defective dystroglycan glycosylation and strongly reduced affinity for its ligands and that mutations in dystrophin main ABD lead to severe forms of dystrophy (Henderson et al., 2010; Kanagawa, 2021). However, as explained in the next paragraphs, the available studies provide alternative explanations for these requirements. Also, dystrophin absence in muscle can be partially rescued by overexpression of alpha7-integrin, a *bona fide* adhesion protein (Burkin et al., 2001). Nevertheless, it would be necessary to show the reverse, i.e., alpha7-integrin absence rescued by DAPC gain of function, to unequivocally demonstrate that this genetic interaction reveals a DAPC cell adhesion function. Moreover, RNAi depletion of *dystrophin* or inducible mutation of *dystroglycan* in fully differentiated mouse muscle fibers has no or very limited impact on muscle structure and function (Ghahramani Seno et al., 2008; Rader et al., 2016). Although partial efficiency of RNAi or perdurance of dystrophin and dystroglycan proteins could mask some effects, these results argue for a developmental function rather than a direct structural maintenance role for the DAPC.

### Where is the DAPC required in muscle cells?

If DAPC role is to mechanically protect muscle cells, directly or indirectly, then an important question is where DAPC is required within muscle cells. The most frequent suggestion is that the DAPC and especially dystrophin are required at costameres, which are the sites of ECM attachment at the Z-disc level at each sarcomere extremities. Costameres were the first site where dystrophin localization was reported (Minetti et al., 1992; Straub et al., 1992). However, there are now several indications that dystrophin is much more concentrated at myotendinous junctions (MTJ), from zebrafish to mammals (Bassett et al., 2003; Bajanca et al., 2015; Ruf-Zamojski et al., 2015; Morin et al., 2023). Accordingly, MTJ structural defects have been reported in several mouse and zebrafish dystrophin mutants (Law and Tidball, 1993; Ridge et al., 1994; Bassett et al., 2003). These defects are rescued by microdystrophin transgenes, suggesting a correlation between these structural defects and muscle degeneration (Banks et al., 2008; Banks et al., 2010). The MTJ is where there is an obvious monaxial tension increase during contraction while force distribution at the sarcolemma ECM interface is less straightforward. For instance, myofiber shortening is associated with an increase of its section area, leading to complex force repartition. Sarcolemma rupture is usually evaluated with cell permeability assays using dyes that normally do not enter the cells and then is visualized in muscle tissue sections. Unfortunately, this does not give information on where the rupture occurs along the myofiber longitudinal axis (i.e., MTJ *versus* lateral sarcolemma). Similar assays in whole mouse (or whole muscle) revealed that the dye is mainly incorporated at the muscle extremities. This suggests that the rupture occurs at or close to MTJ (Straub et al., 1997; Banks et al., 2008). Alpha7-integrin also is mainly concentrated at MTJ, suggesting that it could be the main site of their functional relationship (Mayer et al., 1997). Finally, recent data indicate that the upregulation of ECM genes in specialized nuclei close to MTJ is lost in syncytial skeletal muscle cells from *mdx* mice, although the underlying mechanism is not known (Kim et al., 2020). Besides costameres and MTJ, there is evidence that DAPC is required for the proper assembly of neuromuscular junctions, but this topic will be not developed in this review (Belhasan and Akaaboune, 2020; Lovering et al., 2020). Altogether, the available data suggest that MTJ may be the main site where the DAPC is required.

### Comparison of DAPC with cell adhesion complexes

**FIGURE 4:**
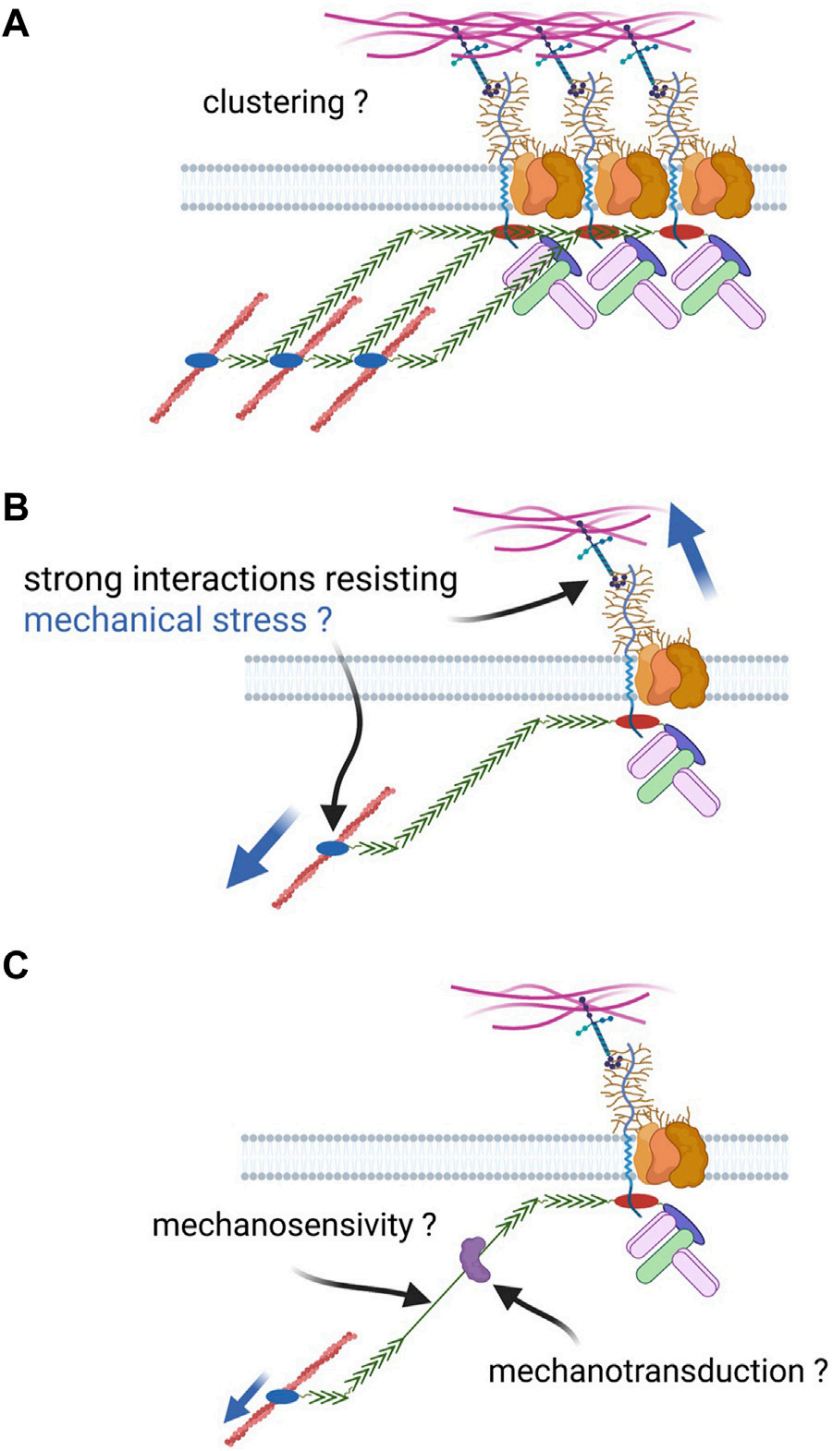
Open questions about DAPC cell adhesion function. Based on the knowledge of well-defined adhesion complexes, different properties could be expected for the DAPC if it had a similar function: **(A)** capacity to form clusters with a cooperative effect on adhesion; **(B)** Strong link with extracellular ligands and the cytoskeleton to resist a strong mechanical tension; such link can be created by molecular catch bonds; **(C)** the complex could be mechanosensitive, for instance through the unfolding of dystrophin spectrin repeats, and allow mechanotransduction by recruiting proteins upon conformational change.

After at least 3 decades of research, a clear demonstration is still missing to link what is observed at the tissue and cell scales to what we know about DAPC at the molecular scale. Our knowledge and understating of a putative DAPC adhesion function are very limited compared with well-defined adhesion complexes. The next paragraphs will compare common features of these adhesion complexes (i.e., cadherin and integrin) with the DAPC (Figure 4).

#### Clustering

Both cadherin and integrin complexes form clusters with a cooperative effect on adhesive properties, as observed at adherens junctions and focal adhesions, respectively (Guillot and Lecuit, 2013; Charras and Yap, 2018; Pinheiro and Bellaïche, 2018; Kechagia et al., 2019; Kanchanawong and Calderwood, 2023). Such clustering relies on the complex component capacity to physical interact with each other. For instance, E-cadherin extracellular domains can interact in *cis* and in *trans*, via the EC1 and EC2 domains, respectively. Conversely, integrin clustering relies more on intracellular mechanisms.

On the other hand, no evidence supports the potential clustering, or even the dimerization, of dystrophin and dystroglycan, the DAPC core components. First, dystroglycan dimerization has never been described and is not expected on the basis of its secondary structure. Second, dystrophin intracellular dimerization has been hypothesized several times, but has never been demonstrated. SR discovery suggested a potential dimerization based on alpha/beta-spectrin interaction in an antiparallel manner. However, self-association of dystrophin SRs has not been observed, and structural data on its rod domain suggest a different SR arrangement compared with spectrins (Delalande et al., 2017). This dissimilarity also suggests that dystrophin works on a different manner than spectrin-actin networks, though it cannot be excluded that it provides a protective effect on cell membrane as spectrins by forming an in-plane network resisting shear stress (Leterrier and Pullarkat, 2022). Moreover, structural data initially suggested that dystrophin actinin-like domain forms dimers, but subsequent work indicated that it was an artefact due to the introduction of point mutations to stabilize the structure (Norwood et al., 2000; Singh and Mallela, 2012). Our current knowledge of the molecular interactions with dystrophin (and also syntrophins, dystrobrevin) does not allow proposing an indirect mechanism for a potential dimerization. In agreement, there is no biochemical evidence of clusters of several DAPC with, for instance, utrophin and dystrophin found in the same complex, and it has been proposed that DAPC works as a monomer (Rybakova and Ervasti, 1997). Lastly, despite its subcellular enrichment at some specific places (e.g., costameres), the idea of DAPC distribution in clusters is not supported by descriptive data, obtained for instance by super-resolution microscopy or electron microscopy.

#### High interaction affinity chain between ligands and cytoskeleton

To maintain cohesiveness, a complex must provide a strong linkage between extracellular ligands and the cytoskeleton that can withstand significant mechanical loads. Such property is even more crucial in muscle than in any other tissue, due to the extreme force generated by the optimized myofiber bioengine. The evolution of DAPC and dystrophin suggests that the link with the ECM is a constant feature, while the link with the actin cytoskeleton appeared later and is not associated with all DAPC forms based, for instance, on dystrophin alternative isoforms. Moreover, dystroglycan has critical functions independently of its intracellular domain (see below). This is an important difference from textbook adhesion complexes the function of which is always dependent on their capacity to maintain the link between cytoskeleton and their ligands.

The main evidence of a strong DAPC-mediated link with F-actin comes from sarcolemma pealing experiments, in which costameric F-actin remains attached to the membrane in wild-type but not in *mdx* muscles (Rybakova et al., 2000). However, this assay was used in a non-quantitative manner and this result may appear surprising because of the compensatory expression of utrophin in *mdx* mice. Subsequent work suggested that this strong link could be mediated by the second ABD of dystrophin (ABD2), within its rod domain, explaining why utrophin does not provide such a strong link (Warner et al., 2002; Hanft et al., 2006). However, according to the same assay, utrophin overexpression in *mdx* muscle restores the strong link between sarcolemma and costameric actin (Rybakova et al., 2002). Moreover, expression of dystrophin transgenes without ABD1 rescues only weakly the *mdx* mouse phenotype, whereas expression of dystrophin transgenes without ABD2 are efficient. This indicates that ABD1 is more important, as suggested also by its better evolutionary conservation (Warner et al., 2002). Intriguingly, analysis of dystrophin features in patients with BDM harboring point mutations in ABD1 suggests that disease severity correlates more with the impact of such mutations on protein stability than on F-actin affinity (Norwood et al., 2000; Henderson et al., 2010). Thus, either actin binding is not instrumental for dystrophin main function, or there is partial redundancy between ABD1 and ABD2. Therefore, it would be interesting to reassess using current biophysical and quantitative imaging assays whether dystrophin provides a strong link to F-actin, through which domain, and more importantly to which extent this is essential for its function.

On the other hand, the mechanisms by which integrin and cadherin complexes establish a strong link with F-actin to withstand mechanical stress are still under extensive investigation. Recent studies have revealed that evolution has solved this complex issue with a remarkable feature shared by both complexes. They display a strength of interaction with F-actin that increases with tension due to force-dependent conformational changes, forming a so-called asymmetric catch bond (Huang et al., 2017; Arbore et al., 2022; Owen et al., 2022; Wang et al., 2022). It is not known whether dystrophin exhibits such a feature. Biochemical approaches have found relatively weak affinity of ABD1 for F-actin, which could be consistent with a catch bond (Rybakova et al., 1996). Interestingly, catch bond features have been recently demonstrated for actinin binding with F-actin, making this property plausible for dystrophin (Hosseini et al., 2020).

At the other extremity of this chain, glycosylated dystroglycan interacts with its ligands through their laminin globular domain. These ligands include laminin, but also other ECM proteins, such as perlecan, agrin and pikachurin (Ibraghimov-Beskrovnaya et al., 1992; Gee et al., 1994; Peng et al., 1998; Sato et al., 2008). Catch bond properties have been also identified for E-cadherin homophilic interactions and for integrin interaction with its ligands (Perret et al., 2004; Kong et al., 2009; Rakshit et al., 2012). It is unknown whether dystroglycan binding to its ligands can be similarly reinforced by tension. One way to investigate whether DAPC provides high adhesion to basal lamina is to impair the glycosylation required for its interaction with its ligands (Han et al., 2009). In such conditions, sarcolemma fragility and detachment of the basal lamina are observed, suggesting a direct link between the adhesion provided by dystroglycan and its protective effect on muscle cells. However, subsequent research showed that glycosylated dystroglycan functions as a scaffold for proper basal lamina assembly in muscles (Goddeeris et al., 2013). Therefore, it is unclear whether the observed detachment is a direct consequence of dystroglycan loss of adhesion properties or an indirect effect due to lamina abnormalities and consequently, weakened integrin-mediated adhesion. Specifically, local modifications of mechanical properties, such as cell stiffness and adhesion force, have never been spatially correlated with DAPC presence at the molecular or even subcellular scale.

#### Mechanosensitivity and mechanotransduction

Both integrin and cadherin complexes are mechanosensitive (i.e., their conformation changes under tension) and are implicated in mechanotransduction, recruiting new actors once in an open conformation (Charras and Yap 2018; Pinheiro and Bellaïche 2018; Kechagia et al., 2019; Kanchanawong and Calderwood 2023). The change in applied tension reveals a binding site for vinculin in alpha-catenin and in talin, in cadherin and integrin complexes, respectively. This recruitment reinforces the mechanical link between adhesion complexes and cytoskeleton as vinculin also binds F-actin. Moreover, mechanotransduction by these complexes is involved in the regulation of important signaling pathways, such as the YAP/Taz-hippo pathway (Cai et al., 2021).

Evidence for dystrophin role as a mechanosensor and for DAPC implication in mechanotransduction are so far indirect (recently reviewed in (Wilson et al., 2022)). For instance, in the absence of dystrophin, the stretch-activated calcium channel TRCP6 is hyperstimulated in cardiomyocytes. This seems critical for the cardiopathy observed in DMD (Fauconnier et al., 2010; Seo et al., 2014; Chung et al., 2017). However, although some channels are recruited by DAPC, there is no established link with TRPC6. Similarly, several studies described changes in the response to a mechanical stress in cells harboring a dystrophin mutation (Pasternak et al., 1995; Lopez et al., 2021; Ramirez et al., 2022). However, in all these examples, it is unclear whether such changes (e.g., TRCP6 hyperactivation) are a direct consequence of DAPC absence/alteration or whether they are a secondary effect of cell/ECM interface disorganization and changes in the cell mechanical properties.

Concerning mechanosensitivity, the usual suspect is the dystrophin rod domain and its SRs. It is now established that spectrins are mechanosensitive proteins (Duan et al., 2018; Lardennois et al., 2019; Mylvaganam et al., 2022). The main evidence concerning dystrophin mechanosensitivity came from elegant and purely *in vitro* approaches showing that the rod domain can be mechanically unfolded, stretching up to 800 nm (Bhasin et al., 2005; Le et al., 2018). Each SR can be unfolded in a range of force between 10 and 30 picoN, although the SR18-24 region is slightly more resistant than the rest of the rod domain. However, in this experimental set-up, the rod domain extremities are artificially bound to substrates. Thus, it is unclear whether, *in vivo,* affinity for F-actin and dystroglycan can support the range of tension to allow rod domain stretching. Finally, if the rod domain acts as a kind of spring, the relevance of its length seems relative. Indeed, experiments based on microdystrophin showed that this domain can be reduced from 24 to 4 SRs without any major functional impact.

If DAPC were mechanosensitive, then mechanotransduction could be considered and some interactions with the rod domain could be mechanoregulated. For instance, tension-dependent nNOS recruitment would be meaningful, because this interaction is important during exercise. However, an experimental set-up to test such mechanoregulation of DAPC interactions has not been developed yet.

It has been suggested that dystroglycan also interacts with the hippo pathway in cardiomyocytes by recruiting its effector, the YAP-Taz transcription factor (Morikawa et al., 2017). The hippo pathway and YAP-Taz localization control are part of mechanotransduction mechanisms in different contexts (Cai et al., 2021). Like dystrophin, YAP-Taz has a WW domain that allows the interaction with specific proline-rich motifs on dystroglycan. However, while one could expect a competitive binding, dystrophin is required for YAP-dystroglycan interaction. Thus, more studies are required to understand the interplay between YAP, dystrophin and dystroglycan and to determine whether this could be part of a mechanotransduction mechanism.

## DAPC developmental functions: an organizer of the cell/ECM interface

DAPC role in muscle cells is well established, particularly its mechanoprotective effect. However, it is not known whether this effect is due to its cell adhesion properties or whether it is a secondary effect. Moreover, during evolution, DAPC has not been always associated with muscle cells and many of its components (or dystrophin paralogs in vertebrates) are expressed in almost all cell types (e.g., neurons, epithelial cells). Therefore, looking at different cell types and animal models might give information on its conserved primary functions. The next paragraphs will describe some of these functions, particularly those linked to development.

### Basement membrane secretion and assembly

As mentioned previously, defective dystroglycan glycosylation induces alterations of the myofiber BM structure. This led to the proposal that dystroglycan works as an anchor at cell surface for its proper assembly (Goddeeris et al., 2013). This aspect of DAPC function reminds many data from developmental studies. The development of mouse embryos in which dystroglycan was knocked out is stopped very early with defects in the Reichert’s membrane, one of the first BMs to be produced in the embryo (Williamson et al., 1997). Embryoid bodies from embryonic stem cells harboring mutated dystroglycan do not assemble a BM. Moreover, dystroglycan is required for proper BM assembly in mouse retina and brain (Henry and Campbell 1998; Satz et al., 2008; Satz et al., 2009). In *Xenopus laevis*, loss of dystroglycan affects BM formation and particularly laminin recruitment during notochord and pronephros development (Bello et al., 2008; Buisson et al., 2014).

Several studies in invertebrates also indicate a role of dystroglycan in BM dynamics. During *Drosophila* oogenesis, dystroglycan is specifically required for the formation of BM fibrils that shape the future embryo (Haigo and Bilder 2011; Cerqueira Campos et al., 2020). In *Drosophila* spermatogenesis, BM is part of the niche allowing germinal stem cell maintenance. In some genetic contexts, an expansion of this niche is observed that depends on the local production of dystroglycan and perlecan and the recruitment of soluble laminin (Tseng et al., 2022). In addition, a recent preprint indicates that dystroglycan is required for the proper lamina organization in the fly retina, at the basal domain of the neuroepithelium (Walther et al., 2022).

Mechanistically, there are still two major open questions on dystroglycan developmental roles related to BM. First, it is not clear whether dystroglycan relies on a scaffolding activity at the cell surface for BM assembly, or whether it may also participate in BM component secretion. Second, it has not been determined whether these roles require other DAPC components, especially dystrophin. Dystroglycan knock-out in the mouse leads to very early embryonic lethality; however, dystroglycan intracellular part is dispensable for mouse development (Williamson et al., 1997; Satz et al., 2009). Accordingly, mice in which both utrophin and dystrophin have been knocked out are viable (Grady et al., 1997). Dystroglycan intracellular domain is also dispensable for proper BM formation in the mouse retina and in *X. laevis* notochord (Satz et al., 2009; Buisson et al., 2014). In all these cases, the function as an anchor at cell surface seems the most likely because dystroglycan cannot be connected to the intracellular machinery. However, intracellular BM accumulation has been observed in embryoid bodies from cells harboring a mutated dystroglycan, suggesting a secretion defect (Henry and Campbell 1998). In other contexts, dystrophin, and consequently the intracellular part of dystroglycan, also are involved. In *Drosophila,* the effect of dystroglycan loss on BM is phenocopied by dystrophin mutants in the retina and ovary (Cerqueira Campos et al., 2020; Walther et al., 2022). During oogenesis, both dystrophin and dystroglycan seem involved in the proper subcellular targeting of the BM protein secretory route required for fibril formation (Isabella and Horne-Badovinac 2016; Cerqueira Campos et al., 2020; Dennis et al., 2023). Importantly, in this case, DAPC impact is more qualitative than quantitative, influencing the ECM supramolecular organization, as easily revealed using GFP-tagged ECM proteins. As these tools are not yet available for mammalian models, it is difficult to determine whether this role is conserved. Moreover, it is unclear whether dystrophin requirement for BM organization indicates a different cellular function for the DAPC (i.e., an implication in BM protein secretion), or whether dystrophin may act by controlling dystroglycan subcellular localization and thereby where and how BM is assembled.

### DAPC roles in tissue morphogenesis

**FIGURE 5:**
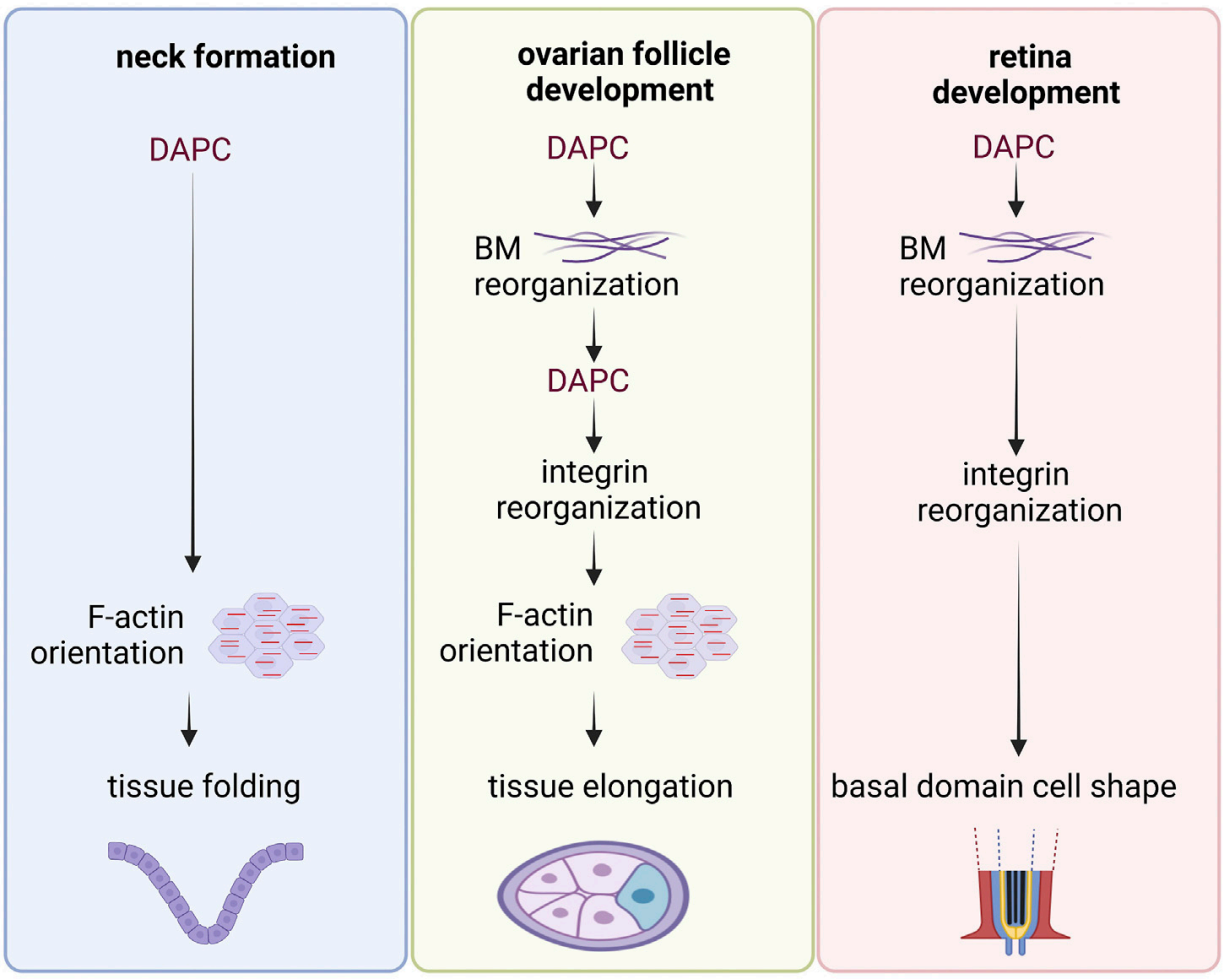
Examples of DAPC involvement in tissue morphogenesis. Common steps can be identified in the sequence of events observed in the different examples of tissue morphogenesis involving the DAPC in *Drosophila*. In each example, the DAPC acts upstream in the mechanism. Its action induces the reorganization of the basement membrane (BM) and/or a preferential orientation of the actin cytoskeleton. Importantly, it is not always known whether DAPC effect is direct or indirect.

DAPC has been implicated in the morphogenesis of different tissues in various model organisms. In flies, this includes ovarian follicle elongation, adult neck formation, and retina development, and in *X. laevis*, notochord and pronephros development (Bello et al., 2008; Buisson et al., 2014; Cerqueira Campos et al., 2020; Walther et al., 2022; Villedieu et al., 2023). Although it is impossible to propose a unifying scheme of DAPC participation in morphogenesis, some shared features can be observed across different systems (Figure 5). First, as previously explained, DAPC can play an essential role in the proper BM organization. BM is then involved in tissue morphogenesis, for instance during fly ovarian follicle elongation and retina development and *X. laevis* notochord and pronephros development. Second, DAPC can promote F-actin reorganization on the basal side of epithelial cells, specifically at the contact with BM, like during follicle elongation and neck formation where DAPC controls the orientation of F-actin fibers (Cerqueira Campos et al., 2020; Villedieu et al., 2023). This preferential orientation at the supracellular scale creates a mechanical strain, like a belt or a corset, that shapes the tissue. During fly ovarian follicle elongation, this function can be dissociated from the one linked to BM fibril formation, although such fibrils are required for actin orientation. In ovarian follicle cells, DAPC effect might be indirect because actin fiber orientation relies on the proper positioning of integrin-dependent focal adhesions. However, it is not known how DAPC can spatially organize focal adhesions in a cell autonomous manner. Importantly, in this tissue, dystroglycan loss does not enhance the global epithelium architecture disorganization caused by integrin loss, suggesting the absence of redundancy concerning adhesion to the BM (Lovegrove et al., 2019). During *X. laevis* notochord development, independently of its impact on BM, dystroglycan is also required for the proper radial cell intercalations, suggesting that it participates in the polarized reorganization of the cytoskeleton (Buisson et al., 2014). This function depends on its intracellular domain, although the mediators are not known. In the fly retina, it has been suggested that DAPC controls where BM is deposited, and this then influences integrin localization. Ultimately, integrins may define the cell shape by controlling actin cytoskeleton. During fly neck formation, it is not known whether DAPC function relies on its effect on BM and/or on integrins or whether it reflects a more direct function in actin organization (Villedieu et al., 2023). Beside these studies showing an impact on cytoskeleton organization, a recent preprint reveals a striking planar polarized pattern requiring DAPC in *C. elegans* muscle cells, suggesting that subcellular polarized targeting might be a general feature for this complex (Peysson et al., 2023). Overall, a first set of morphogenetic events controlled by dystroglycan are linked to its involvement in BM assembly, whereas a second set orients the cell cytoskeleton reorganization, although both can coexist with some interdependence in the same tissue.

## Conclusion

This review shows that the DAPC is very conserved during metazoan evolution, but that its history is not necessarily linked to that of muscle cells. Interestingly, the conservation of poorly characterized domains of the large dystrophin protein suggests that our current knowledge about its function is still incomplete. Moreover, careful analysis of the available data does not allow saying that the DAPC corresponds to a proper cell adhesion complex. Its roles during development rather suggest a function as an organizer of the ECM/F-actin interface. More studies are needed to reach a more unified view of its functions at the molecular, cellular and tissue scales in normal and pathological contexts.
